# Asteroid Saponins: A Review of Their Bioactivity and Selective Cytotoxicity

**DOI:** 10.3390/md22120552

**Published:** 2024-12-07

**Authors:** Stuart J. Smith, Tianfang Wang, Scott F. Cummins

**Affiliations:** 1 Centre for Bioinnovation, University of the Sunshine Coast, Maroochydore BC, QLD 4558, Australia; ssmith16@usc.edu.au (S.J.S.); twang@usc.edu.au (T.W.); 2 School of Science, Technology and Engineering, University of the Sunshine Coast, Maroochydore BC, QLD 4558, Australia

**Keywords:** asteroids, bioactivity, natural products, saponins

## Abstract

Saponins are a diverse class of secondary metabolites that are often reported to exhibit a variety of pharmacological applications. While research into the elucidation and application of plant and class Holothuroidea-derived saponins (i.e., sea cucumbers) is extensive, the class Asteroidea-derived saponins (i.e., seastars) have been largely overlooked and primarily limited to elucidation. This review provides a comprehensive overview of the cytotoxic activities of asteroid-derived saponins against various cell cultures, for instance, mammalian erythrocytes, multiple microbial strains and cancer cell lines, including melanoma, breast, colon, and lung cancers. Highlighting the distinct structural variations in these saponins, this review examines their selective cytotoxicity and potency, with many demonstrating IC_50_ values in the low micromolar range. Specific compounds, such as asterosaponins and polyhydroxylated saponins, exhibit noteworthy effects, particularly against melanoma and lung carcinoma cells, while triterpenoid saponins were found to be highly cytotoxic to both erythrocytes and fungal cells. This review also addresses gaps in the research area, including the need for additional in vitro antimicrobial studies, in vivo studies, and further exploration of their mechanisms of action. By consolidating recent findings, we have shed light on the therapeutic potential of asteroid-derived steroidal saponins in developing novel antimicrobial and anticancer agents.

## 1. Introduction

Saponins are secondary metabolites predominantly found in plants, though their presence in animals is comparatively rare and limited to certain marine invertebrates. Among those marine invertebrates, saponin production is primarily observed in three animal classes, namely the Holothuroidea and Asteroidea (both phylum Echinodermata), as well as the sponge class Demospongiae (phylum Porifera) [1,2,3,4]. Numerous studies have been conducted to characterising the large chemical diversity of marine saponins; however, only recently has the focus on pharmacological activities been applied [4].

Asteroids, or seastars, are particularly unique within Echinodermata due to their production of a distinct class of saponins known as asterosaponins, alongside other polyhydroxylated steroidal saponins [4,5]. The biological role of these compounds in asteroids remains speculative; however, their steroidal glycosides have been commonly considered to be significant contributors to their toxicity and the main agents responsible for their diverse pharmacological effects, including cytotoxicity, haemolysis, as well as antibacterial, antifungal, and antiviral properties [4,5,6,7,8]. In plants, steroidal saponins are known to be widespread throughout traditional Chinese medicine, where extensive studies of saponins with either steroidal or triterpenoid structures have been performed, demonstrating their natural roles as antifouling agents, acting as chemical defences against microbial infections and natural insecticides [9,10,11].

The incubation of cells and tissues with saponin has been found to make the cell lipid bilayer permeable, with saponin-lysed erythrocytes failing to reseal, indicating irreversible damage [12]. Given their membranolytic and haemolytic properties, saponins are known to be toxic to vertebrate predators. Pathological investigations with both plant and holothuroid-derived saponin extracts have determined organ-specific, dosage-dependent responses, with both structural and functional losses observed when exposed to sub-lethal quantities of saponins [13,14,15,16]. As such, any potential pharmaceutical applications should demonstrate high efficacy against target cells while minimising harm to normal cells. To address the haemolytic factor for saponins, erythrocytes are commonly used as models to assess the haemolytic potential, with both mouse and human red blood cells being standard models for these studies [17,18]. Steroidal saponins are believed to block rapid cellular proliferation by regulating the mitotic cell cycle, and in addition, they are reported to potentially trigger both extrinsic and intrinsic apoptosis pathways associated with tumour promotor inhabitation [19].

The application of saponins is subject to regulatory oversight when used in pharmaceuticals, food, or cosmetics. In the U.S., the FDA regulates saponin-containing products, requiring rigorous safety and efficacy evaluations before approval for pharmaceutical use [20], while the European Medicines Agency oversees similar processes in the EU [21]. Commercially, saponins hold promise, particularly in the natural product, supplement, and nutraceutical markets, due to their potential health benefits, including anticancer, anti-inflammatory, and immune-modulating effects [22,23,24]. However, their viability in the pharmaceutical sector depends on overcoming challenges related to bioavailability, toxicity, and large-scale production. Saponins are valued for their surfactant properties in food and cosmetics, though their use must comply with food safety and cosmetic regulations [25,26]. The growing market for natural health products enhances their commercial potential, particularly in functional foods and natural therapies [22,27].

Recent studies have isolated and characterised asteroid saponins with triterpenoid aglycones, structures typically found in holothuroids and demosponges [15,28]. It remains unclear whether these saponins are fully synthesised by the asteroid individual themselves, or acquired from their diet, as their structure shares characteristics with sea cucumber glycosides. Despite this, the potential applications of these saponins have been demonstrated in several studies, highlighting their promising bioactivity [15,28]. A previous review published in 2019 investigated the potential antitumour action of asteroid-derived compounds [29]. Utilising a portion of the same studies as this review, their focus was on the wider variety of compounds found in individual species. Within that review, a summary of identified compounds was provided; however, little chemical information was given; therefore, comparative evaluations could not easily be made to determine any specific saponin-to-cell line combination effectiveness [29]. With the recent release of the Marine Animal Saponin Database [30], there exists a resource that succinctly categorises known saponin structures by structural characteristics and taxonomic origin, providing the original elucidation publication for each saponin [30]. Within these chemical elucidation publications, many contain quantitative application testing of isolated saponins, thereby providing valuable data for comparison [30].

This review aimed to analyse the available quantitative data on in vitro effectiveness of saponins isolated from asteroids. It focused on compiling and analysing studies that investigate the bioactivity of these marine-derived compounds as haemolytic agents in addition to their cytotoxic effect against several bacterial strains, fungi cultures, and various cancer cell lines, providing insights into their mechanisms of action, effectiveness, and potential therapeutic applications.

## 2. Results

### 2.1. Overview

Applying the specified search parameters to the MASD v1.0 [30] reduced the number of relevant publications from 345 to 34 studies. These selected studies reported quantitative data from cytotoxicity tests conducted on various cell cultures, including bacterial and fungal strains, mammalian erythrocytes and splenocytes, as well as cancer cell lines. All bioassays utilised saponins isolated from asteroids. The following is an overview of the outcomes derived from these studies, summarising key findings and highlighting the biological activity of asteroid-derived saponins. Variability in the potency of the saponins was observed across different species, assay types, and target cells. A full tabulation of each study’s bioactivity results is detailed in File S1.

### 2.2. Asteroid-Derived Saponin Haemolytic Activity

Haemolytic activity, a common characteristic of many saponins, reflects their ability to disrupt cell membranes, particularly red blood cells, leading to haemoglobin release [31]. This property is closely linked to their amphiphilic structure, which enables them to interact with lipid bilayers. Understanding the haemolytic potential of saponins is crucial, not only for determining their toxicity, but also for exploring their therapeutic window for safe pharmaceutical application.

Six studies assessed the haemolytic activity of 23 asteroid-derived saponins on erythrocytes from three mammalian species. One study investigated the haemolytic activity of nine triterpenoid saponins (pacificusosides D-K and cucumarioside D) isolated from the *Solaster pacificus* on human erythrocytes [15]. The results revealed that pacificusosides D, F, and H, along with cucumarioside D, exhibited high cytotoxicity, with effective dose 50 (ED50) values of 2.03, 0.72, 1.68, and 2.48 µM, respectively [15]. In another study, three unnamed asterosaponins (referred to as saponin 1, 2, and 3 in the paper) isolated from *Culcita novaguineae* were tested for haemolytic activity using rabbit erythrocytes [7]. Saponin 1 and 3 demonstrated moderately high haemolytic activity with ED_50_ concentrations of 16 and 31 µg/mL, respectively, while Saponin 2 was found to be inactive at concentrations below 40 µg/mL [7]. Additionally, three steroidal saponins (aphelasteroside C, cheliferoside L1, and forbeside E3) isolated from *Aphelasterias japonica* were tested on mouse erythrocytes [32]. These saponins exhibited mild haemolytic activity, with ED_50_ values of 190, 175, and 330 µM, respectively [32].

A separate study examined the cytotoxic effects of 13 steroidal saponins isolated from an ethanolic extract of *Choriaster granulantus* on splenocytes from CD-1 mice [33]. Of these, only four of the saponins displayed notable cytotoxic activity, with laeviuscoloside D being the most potent (IC_50_ = 2.20 µM), followed by granulatoside D, echinasteroside F, and desulphated echinasteroside B, which had IC_50_ values of 4.70, 4.60, and 4.50 µM, respectively [33]. This might be attributed to glycosylation patterns, hydrophobicity, and steric effects. In plants, active saponins predominately feature mono- or disaccharide substitutions on the aglycone backbone, which may enhance their hydrophilic–lipophilic balance and interactions with cellular targets, while inactive saponins often lack such substitutions or possess less favourable structural configurations. The close IC50 values among the active saponins indicate a likely shared mechanism of action, such as membrane disruption or specific receptor interactions, although further studies are needed to fully elucidate these structure-activity relationships.

In another investigation, ten polyhydroxysteroidal saponins (anthenosides L-U) were isolated from *Anthenea aspera* and tested on CD-1 mouse erythrocytes [34]. While most saponins showed limited activity, a mixture of anthenosides T and U, which could not be separated by repeated reversed-phase HPLC, exhibited significant haemolytic activity with an EC_50_ value of 8 µM [34]. Finally, four steroidal saponins (henricioside H2, laeviusculoside A, J, and G) isolated from the water-soluble fraction of an ethanolic extract from *Henricia leviuscula* were tested on mouse erythrocytes [35]. Henricioside H2, laeviusculoside J, and G demonstrated haemolytic activity with half-maximal haemolytic concentration (HC50) values of 120, 130, and 80 µM, respectively, while laeviusculoside A showed no haemolytic activity at the concentrations tested [35].

The bioactivity tests utilising erythrocytes and splenocytes indicated that saponin haemolytic factor varies widely depending on the structure. The structures that show the greatest haemolytic bioactivity were the nine triterpenoid saponins isolated from *S. pacificus* and the four bioactive steroidal saponins from *C. granulantus*, both demonstrating extreme bioactivity to erythrocytes and splenocytes [15,33]. These saponins all achieved haemolytic activity at below 10 µM with distinctly different structures, the triterpenoid saponins derived from *S. pacificus* are tetrasaccharides, while the steroidal saponins from *C. granulantus* are mono and disaccharides. Conversely, the other three studies found that steroidal saponins exhibited moderate to weak haemolytic activity, indicating potential for non-haemolytic saponins [7,32,34,35].

In summary, these studies have provided valuable insights into species-specific differences in erythrocyte susceptibility, variation in saponin structures, and the concentration-dependent nature of haemolysis, offering critical data for balancing cytotoxicity and therapeutic benefits in future drug development. Additionally, these findings inform the design of saponin-based formulations with reduced side-effects, aiming to harness their bioactive potential while minimising risks.

### 2.3. Asteroid-Derived Saponin Antimicrobial Activity and Applications

Research into the antimicrobial properties of asteroid-derived saponins remains limited, with only a few studies providing quantitative data on their efficacy. Specifically, three studies have investigated the antimicrobial potential of these compounds, one focusing on bacterial strains, while the two other studies targeted the fungal species *Pyricularia oryzae*. In total, 11 saponins isolated from two asteroid species have been tested against one fungal species and three bacterial strains.

In the first study, four steroidal saponins (certonardosides K–N) were isolated from the bioactive fractions of an *Certonardoa semiregularis* extract [36]. These saponins were subsequently evaluated for antibacterial activity against 20 clinically isolated strains. Certonardosides K–M displayed limited bioactivity against *Streptococcus pyogenes* 308A and *Pseudomonas aeruginosa* 1771 and 1771M, achieving minimum inhibitory concentrations (MIC) at the highest tested concentration of 25 µM. Similarly, Certonardoside N exhibited MIC values of 25 µM against both strains of *Pseudomonas aeruginosa* but was inactive against *Streptococcus pyogenes* 308A at the tested concentrations. Notably, the remaining 17 unspecified strains tested showed no inhibition at the concentrations examined [36].

In two other studies, *P. oryzae*, a plant pathogenic fungus, was selected as the test organism for the initial screening of antifungal agents. Two n-BuOH extracts, derived from bioassay-guided fractionations of *Culcita novaeguineae*, showed a significant deforming effect on *P. oryzae*, leading to the isolation of four novel and unnamed asterosaponins (referred to as asterosaponin 1 and saponin 1–3 in the study), alongside three known compounds (thornasteroside A, marthasteroside A1, and regularoside A) [7,8]. When these asterosaponins were tested for their ability to induce morphological deformation of *P. oryzae* mycelia, six out of the seven exhibited notable activity, with minimum morphological deformation concentration (MMDC) values ranging from 4 to 64 µg/mL. Among these, saponin 2 (C_49_H_77_NaO_26_S) demonstrated the weakest antifungal bioactivity, with an MMDC value of 256 µg/mL [7,8].

In summary, saponins, due to their amphiphilic nature, can disrupt microbial cell membranes, potentially offering broad-spectrum activity. However, the limited data available points to the need for further exploration of their mechanism of action, spectrum of activity, and the potential for development of resistance. Expanding this research could help elucidate the full antimicrobial potential of asteroid-derived saponins, paving the way for their use in combating bacterial and fungal infections, particularly in the context of rising antimicrobial resistance. Despite the small scope of this research, these findings suggest a promising yet largely unexplored avenue for the development of novel antimicrobial agents.

### 2.4. Asteroid-Derived Saponin Anticancer Agents

Thirty studies have assessed the cytotoxic effects of 67 saponins isolated from asteroid species, with 16 saponins being tested in multiple studies. The inhibitory concentration (IC_50_) of each saponin was determined through the use of one of three variants of colorimetric cell viability assay against 33 different cancer lines, wherein each cell line colony was incubated with the test saponin for a period of between 6 and 120 h. For a detailed synopsis of these asteroid saponins in anticancer, refer to File S2. These saponins demonstrated potential for significant bioactivity against cancer cells, achieving inhibitory effects at sub-micromolar concentrations. However, the activity was selective, with certain cancer cell lines resisting saponin exposure. The structural diversity of the saponins also resulted in varying levels of effectiveness, with some isolates failing to achieve any inhibitory action.

In summary, Halityloside D, a disaccharide steroidal saponin isolated from three asteroid species–*A. carinifera*, *C. semiregularis*, and *C. novaeguineae*–emerged as the most frequently tested saponin in vitro. It was evaluated for cytotoxic effect against 11 different cell lines (Table 1), demonstrating moderate cytotoxicity overall. The most notable bioactivity was observed against the human prostate cancer cell line LNCaP, with an IC_50_ value of 31.80 µM concentrations [37]. However, due to the lack of testing on non-tumorigenic cells, its potential cytotoxicity against normal cell types remains unknown. On the other hand, the RPMI-7951 melanoma cell line emerged as the most widely tested cell line, with a total of 48 saponins tested for cytotoxic effects across ten studies (Table 2). Among these, cucumarioside A10, a triterpenoid saponin isolated from the far eastern seastar *Solaster pacificus* demonstrated the most potent cytotoxic activity against RPMI-7951, achieving an IC_50_ value of 5.80 µM [28]. This highlights the promising potential of certain asteroid saponins as effective anticancer agents, particularly against melanoma, and underscores the need for further investigation into their therapeutic applications.

JB6 Cl41 was a unique cell line among the bioassays conducted, as it is derived from non-tumorigenic epidermal cells from CD-1 mice. As the only non-tumorigenic cell line tested, JB6 Cl41 is the current indicator available for determining saponins cytotoxicity to normal cells. The triterpenoid saponins isolated from S. pacificus demonstrated high bioactivity against JB6 Cl41, with nine out of ten of the lowest IC50 concentrations (Table 3). This is consistent with the haemolytic results discussed previously, indicating structural characteristics that are highly cytotoxic to non-cancerous cells. The seven most bioactive saponins exhibit minimal aglycone structural variation, consisting of the same triterpenoid aglycone, with the only significant structural variance existing in the saccharide moieties attached at C3. Therefore, a conclusion can be drawn that the triterpenoid Pacificusoside saponins derived from S. pacificus are non-viable for human application as the risk of non-selective toxicity is high.

Alternatively, Versicoside A, Aphelasteroside A, and Asterone Thornasteroside A demonstrated weak to no cytotoxic effect to JB6 Cl41; however, no significant distinction was demonstrated between cell lines in all tests conducted with these saponins, further testing would be required to identify viable applications; currently, there is no evidence of any significant bioactivity. Conversely, Regularoside A warrants further investigation, demonstrating no cytotoxic effect on JB6 Cl41 cell at concentrations below 50 µM, while exhibiting significant effect against both K-562 and BEL-7402 cell lines at 8.17 and 12.16 µM (IC_50_), respectively (File S1), demonstrating selectivity toward effecting some cancer cells over normal cells [8].

#### 2.4.1. Saponin Susceptible Cancer Cells

The combined studies reviewed here suggest that the BEL-7402 hepatoma and K-562 leukaemia are highly susceptible to saponin exposure. Cytotoxicity tests on these two cell lines consistently demonstrated high bioactivity, with most saponins tested achieving IC_50_ values below 20 µM in each case. Notably, several saponins, including Regularoside A, have also been evaluated against non-tumorigenic cells, allowing for comparative analyses of cytotoxicity. This enables researchers to assess the therapeutic potential of these saponins by comparing IC50 ratios between cancerous and normal cell types, offering insights into their selectivity and safety profiles.

#### 2.4.2. Cell Line Dependent Cytotoxicity

Thornasteroside A (a steroidal pentasaccharide) is an exemplar of how the cytotoxic effect of a compound is dependent on the cell type tested, demonstrating IC_50_ values ranging between 7.39 and 94 µM (Table 4) across a wide array of cell types. Variations in IC_50_ values between tissue types are expected due to structural variations and metabolic differences; however, large variations are seen between cancer forms within the same tissue (e.g., breast or colorectal). This is likely due to variations in the gene mutation combinations present in the cancer cell lines. Similar cell-type-dependent trends have been reported for other saponin derivatives, such as cucumarioside A10 and certonardoside J3 and P1. Cucumarioside A10 exhibited potent activity against RPMI-7951 melanoma cells (IC50 = 5.80 µM) but weaker effects on other cell lines [28], while certonardoside P1 showed higher cytotoxicity against SK-MEL-2 cells (IC50 = 0.40 µM) compared to XF498 (IC50 = 0.54 µM) and HCT15 (IC50 = 3.38 µM). These findings suggest that specific structural features of saponins influence their interactions with cellular components, contributing to selective cytotoxicity. This cell-type dependence underscores the importance of understanding the structural and molecular determinants of saponin activity, as well as the genetic and metabolic variability of target cells.

#### 2.4.3. Structure-Dependent Cytotoxicity

For plant-derived saponins, a structure-function relationship has been proposed, suggesting an inverse correlation between cancer cytotoxicity and the number of sugars present, potentially due to changes in hydrophobicity [58,59]. However, ginseng-derived saponins with varying saccharide counts have shown selective inhibition of cancer cell lines, indicating that specific saccharide linkages or cell line-specific factors may play a role [59]. For marine invertebrate-derived saponins, a clear structure-function relationship has yet to be established, though cell line-specific cytotoxicity has been observed. For example, thornasteroside A exhibited varied effects across different cell lines, highlighting the variable effects of chemical structure in saponin bioactivity. Pacificusoside E-J saponins demonstrated significant cytotoxicity (see Table 3), despite exhibiting polysaccharide structures, indicating a structure-function relationship contrary to that proposed for plants; currently, the majority of asteroid-derived saponins display cell-line-dependent variability similar to thornasteroside A. Due to the limited number of studies testing asteroid-derived saponins across a broad range of cancer cell lines (see Table 1), a comprehensive understanding of the relationship between chemical structural motifs and cancer cell line susceptibility remains elusive.

#### 2.4.4. Synergistic Anticancer Applications

Frondoside A, a triterpenoid saponin isolated from the sea cucumber *Cucumaria frondosa*, was combined with the cancer drugs oxaliplatin and 5-fluorouracil at a ratio of between 1:10 and 1:20 for in vitro testing against HT-29, HCT-116, and HCT-8 colon cancer cell lines [60]. This combination demonstrated a concentration-dependent decrease in cancer cell populations, with IC_50_ values of 0.5, 0.75, and 0.75µM for the HT-29, HCT-116, and HCT-8 cancer cell lines, respectively [60]. The combination of established cancer drugs and Frondoside A proved to be distinctly more potent than the most active asteroid-derived saponins against these cancer cell lines (File S1). The enhanced efficacy of this triterpenoid saponin, particularly when combined with standard chemotherapy drugs, highlights a potential avenue for asteroid-derived saponins as secondary agents in cancer treatment. Furthermore, synergistic effects have also been observed when combining bioactive saponins. Athenoside mixtures, derived from *A. chinensis* and *A. aspera*, showed higher bioactivity than purified individual athenoside saponins [34,55]. These findings suggest that combining saponins, or using them in conjunction with existing cancer therapies, could enhance therapeutic efficacy while reducing required dosages, thereby minimising collateral damage to normal cells, such as erythrocytes.

### 2.5. Multi-Assay Testing for Asteroid-Derived Saponins

Only three saponins, all isolated from *Culcita novaguineae*, were tested across all three categories of haemolytic, antimicrobial and anticancer effects (Table 5) [7]. Saponins 1 and 3 demonstrated promising bioactivity, achieving both cancer cell IC_50_ values and antifungal MMDC at levels lower than the effective dose (ED_50_) for haemolysis. In contrast, Saponin 2 demonstrated limited bioactivity, showing no haemolytic or anticancer effects, and only minimal antifungal activity, with concentrations greater than 220 µM required for inhibition [7].

As evident from the structures (Table 5), the only distinguishing structural variation to Saponin 2 is the cropped alkyl chain on the steroidal head, indicating the change to hydrophobicity is critical to general bioactivity. Saponins 3 demonstrated the greatest potential for use against both K-562 leukaemia and BEL-7402 hepatoma, achieving greater than 8:1 IC_50_ ratios to the erythrocytes tested in both cases; however, the pharmacokinetics of Saponin 3 and its effect on normal liver cells is currently unknown, requiring further bioassay testing before larger scale tests could be advised.

## 3. Discussion

Currently, limitations exist to the economic viability of utilising asteroid-derived compounds as bioactive natural compounds, primarily due to their natural abundance and difficulty in artificial synthesis. Asteroids produce a wide diversity of saponins, resulting in a complex mixture of compounds with a restricted quantity of each individual saponin produced per individual animal. While the non-species specificity of saponins mitigates this issue, as evident with halityloside D and Thornasteroside A, optimising the quantity derived per unit, will require further development of sample preparation techniques. Additionally, their complex molecular structures and heavy molecular weights pose significant challenges for industrial synthesis [61,62]. However, while a synthetic pathway for asteroid-derived saponins is currently unknown, completed biosynthetic pathways have been elucidated for both multiple Holothuroid and plant species, indicating the potential for an asteroid-derived pathway to follow [63,64,65,66].

A small array of saponins have been tested for cytotoxicity against normal cells, currently restricted to JB6 Cl41 mouse cells and a limited number of erythrocyte studies. Further testing of asteroid-derived saponins against non-tumorigenic cells is required to determine their efficacy as pharmaceutical treatment options in antimicrobial or anticancer applications. The study of saponins derived from asteroids has revealed a promising avenue for the development of novel bioactive compounds with potential applications in anticancer, antimicrobial, and other therapeutic areas. However, despite the significant progress made, much remains unexplored. Future research should focus on expanding the spectrum of saponins tested across a wider range of biological activities, including more in-depth studies on their mechanisms of action.

Additionally, greater emphasis should be placed on improving the scalability and sustainable sourcing of asteroid saponins for commercial use. Advancements in synthetic biology and biotechnology could facilitate the production of these bioactive compounds in larger quantities, enhancing their commercial viability. Moreover, exploring synergistic effects between asteroid saponins and existing pharmaceuticals could unlock new therapeutic strategies, particularly in cancer treatment and overcoming drug resistance. As regulatory frameworks for marine-derived bioactives evolve, it will be crucial to address challenges in clinical translation, ensuring that asteroid saponins meet safety, efficacy, and regulatory standards.

## 4. Materials and Methods

### 4.1. Data Collection

Publications were sourced from the Marine Animal Saponin Database V.1.0 [30]. The initial step involved filtering publications based on taxonomic classification, specifically focusing on species within the Asteroidea class. To identify relevant studies that included bioactivity assays, keyword searches were conducted using “bioactiv”, “cytotoxic”, and “anti” within the titles and abstracts. Each identified study was manually reviewed to confirm the inclusion of quantitative bioactivity data. To be included in this review, studies were required to report the isolation and elucidation of a specific saponin from the asteroid specimen under investigation. Studies that only provided data on crude extracts without identifying individual saponins were excluded from the analysis.

### 4.2. Data Analysis

The selected studies reported bioactivity data in either micromolar (µM) or micrograms per millilitre (µg/mL) concentrations. To facilitate accurate comparisons across studies, unit conversions were performed as needed for each saponin and corresponding bioassay result. This ensured consistency and allowed for a more precise evaluation of the bioactivity data across the different studies.

## 5. Conclusions

In conclusion, existing research on asteroid-derived saponins has demonstrated their significant bioactivity and potential for use in anticancer pharmaceutical applications. However, further investigations are necessary to assess their safety, cytotoxic effects, and specific cell line selectivity. This review highlights the limited scope of quantitative application studies on the therapeutic applications of asteroid saponins, revealing a gap in comprehensive efficacy testing across a wider range of cancer types. Additionally, the reliance on sources from the MASD v1.0 constrained this review to a focus on elucidation studies, underscoring the need for broader exploration of these promising bioactive compounds.

## Figures and Tables

**Table 1:** Eight most tested saponins for in vitro cytotoxicity.

Saponin	Cell Line Tested	Study No.	References
Halityloside D	11	3	[37,38,39]
Thornasteroside A	10	3	[8,40,41]
Maculatoside	8	2	[42,43]
Halityloside A	8	2	[37,39]
Halityloside B	8	2	[37,39]
Certonardoside A	8	2	[36,38]
Certonardoside C	8	2	[36,38]
Certonardoside H	8	2	[36,38]

**Table 2:** Twelve cancer cell lines most widely tested against asteroid-derived saponins.

Cell Line	Organism	Tested Saponins	Study No.	References
RPMI-7951	*Homo sapiens*	48	10	[15,28,39,41,42,44,45,46,47,48]
SK-MEL-2		44	8	[15,36,37,43,49,50,51,52]
A549		30	5	[36,49,50,51,53]
SK-OV-3		27	4	[36,49,50,51]
XF498		27	4	[36,49,50,51]
HCT15		27	4	[36,49,50,51]
T-47D		25	6	[39,41,44,45,47,48]
BEL-7402		22	7	[6,7,8,53,54,55,56]
K-562		19	6	[3,4,5,22,29,30]
HCT-116		16	4	[39,41,44,45]
HT-29		14	3	[28,40,42]
JB6 Cl41	*Mus musculus*	16	3	[15,40,57]

**Table 3:** IC_50_ (µM) concentrations for saponins tested against the *Mus musculus* JB6 Cl41 normal epithelial cell line.

Saponin	Chemical Formula	Structure	Concentration (µM)	Cell Viability Method	Incubation Period (h)	Ref
Pacificusoside F	C_55_H_83_NaO_25_S	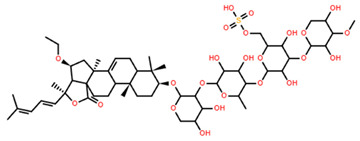	6.00	MTS	24	[15]
Cucumarioside D	C_61_H_94_O_27_	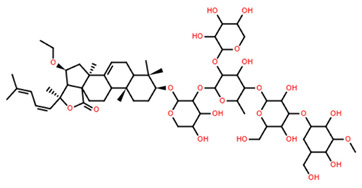	6.10	MTS	24	[15]
Pacificusoside D	C_61_H_94_O_27_	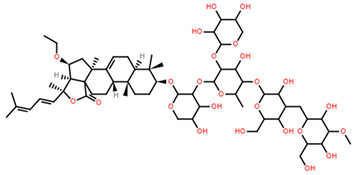	6.40	MTS	24	[15]
Pacificusoside H	C_55_H_83_NaO_26_S	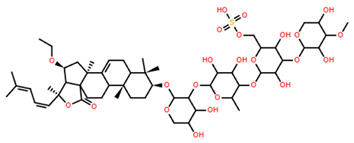	6.60	MTS	24	[15]
Pacificusoside K	C_48_H_73_NaO_23_S	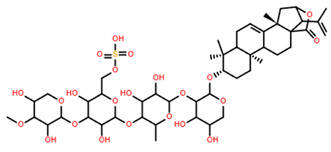	7.40	MTS	24	[15]
Pacificusoside G	C_54_H_82_O_22_	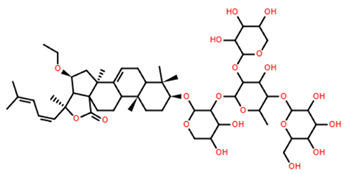	8.70	MTS	24	[15]
Pacificusoside I	C_54_H_84_O_26_	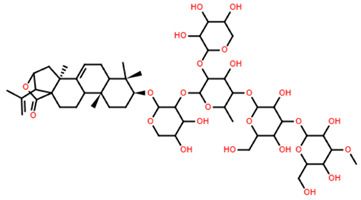	8.70	MTS	24	[15]
Archasteroside B	C_57_H_95_NaO_27_S	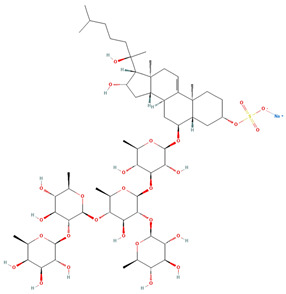	18.00	MTS	6	[57]
Pacificusoside E	C_54_H_82_O_22_	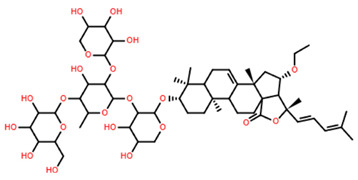	31.50	MTS	24	[15]
Pacificusoside J	C_47_H_72_O_20_	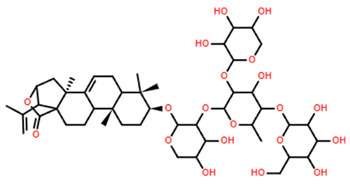	32.80	MTS	24	[15]
Thornasteroside A	C_56_H_91_NaO_28_S	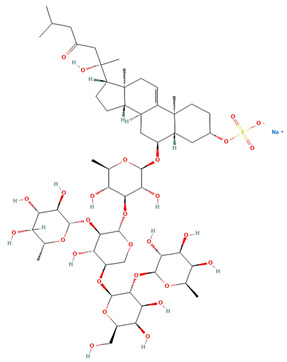	34.00	MTS	24	[40]
Archasteroside A	C_59_H_97_NaO_28_S	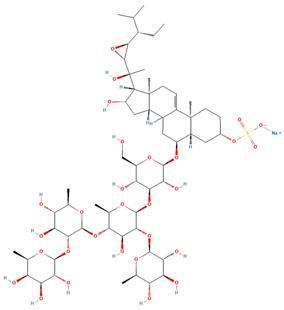	37.00	MTS	6	[57]
Versicoside A	C_62_H_101_NaO_33_S	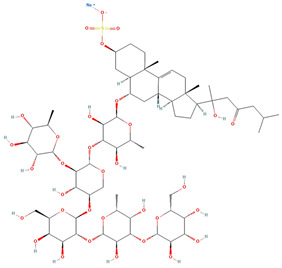	69.00	MTS	24	[40]
Regularoside A	C_58_H_95_NaO_28_S	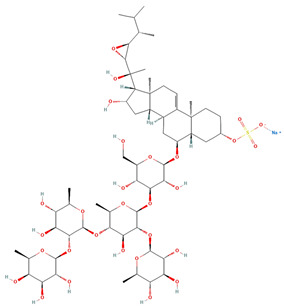	>50	MTS	6	[57]
Aphelasteroside A	C_32_H_55_NaO_12_S	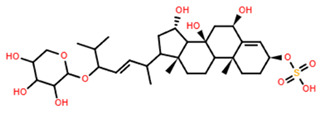	>100	MTS	24	[40]
Asterone Thornasteroside A	C_50_H_79_NaO_27_S	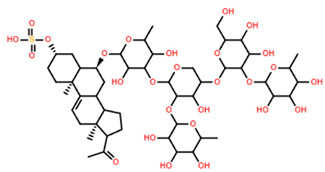	>100	MTS	24	[40]

**Table 4:** IC_50_ (µM) concentration of thornasteroside A (structure shown in Table 3) cytotoxicity tests.

Cell Line	Cell Type Description	Concentration (µM)	Cell Viability Method	Incubation Period (h)	Reference
BEL-7402	human hepatoma	7.39 *	SRB	48	[8]
K-562	human leukaemia	10.61 *	MTT	48	[8]
MDA-MB-231	human breast	28.00	MTS	24	[40]
HT-29	human colorectal carcinoma	32.00	MTS	24	[40]
JB6 CI41	non-cancerous mouse epidermal	34.00	MTS	24	[40]
THP-1	human monocytic leukaemia	39.00	MTS	24	[40]
Raji	human Burkitt’s lymphoma	48.00	MTS	24	[40]
RPMI-7951	human melanoma	50.00	MTS	24	[41]
HCT-116	human colorectal carcinoma	82.00	MTS	24	[41]
T-47D	human breast	94.00	MTS	24	[41]

* Note: converted units from µg/mL.

**Table 5:** Collated bioactivity results for Saponins 1–3, elucidated from a *C. novaguineae* isolation [7].

Saponin	Structure	Cell Line	Measure	Concentration (µM)
Saponin 1 (C_55_H_89_NaO_27_S)	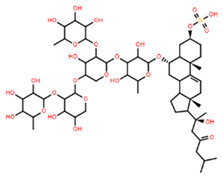	Erythrocyte	ED_50_	12.79 *
		*Pyricularia oryzae*	MMDC	6.39 *
		K-562 ^(1)^	IC_50_	2.85 *
		BEL-7402 ^(2)^	IC_50_	2.04 *
Saponin 2 (C_49_H_77_NaO_26_S)	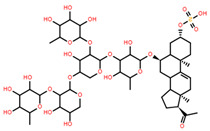	Erythrocyte	ED_50_	>34.75 *
		*Pyricularia oryzae*	MMDC	222.38 *
		K-562 ^(1)^	IC_50_	>43.4 *
		BEL-7402 ^(2)^	IC_50_	>43.4 *
Saponin 3 (C_56_H_91_NaO_27_S)	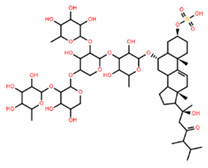	Erythrocyte	ED_50_	24.50 *
		*Pyricularia oryzae*	MMDC	3.16 *
		K-562 ^(1)^	IC_50_	2.96 *
		BEL-7402 ^(2)^	IC_50_	1.49 *

* Note: converted units from µg/mL. ^(1)^ Method: MTT colorimetric assay, 48 h incubation. ^(2)^ Method: SRB colorimetric assay, 48 h incubation.

## Supplementary Materials

The following supporting information can be downloaded at: https://www.mdpi.com/article/10.3390/md22120552/s1, File S1: Case study raw data; File S2: Synopsis of cancer cell line studies; references [6,7,8,15,28,36,37,38,39,40,41,42,43,44,45,46,47,48,49,50,51,52,53,54,55,56,57].
