# Supplementary material for: Asteroid Saponins: A Review of Their Bioactivity and Selective Cytotoxicity
Source: Mar Drugs. 2024 Dec 7;22(12):552. doi: 10.3390/md22120552 (PMC11679803; doi:10.3390/md22120552)
Supplement: Supplementary file 1 [file marinedrugs-22-00552-s001.zip › File S2.pdf]

Synopsis of cancer cell line studies

In vitro cytotoxicity of ten asterosaponins extracted from *Culcita novaeguineae* was evaluated against human leukemia K-562 and human hepatoma BEL-7402 cell lines[6-8]. Among these, three saponins (regularoside B, novaeguinoside I and II) were isolated from ethanolic extracts, while unnamed saponins (saponins 1–3) were obtained through bioassay-guided fractionation of an n-BuOH extract. Four additional asterosaponins (asterosaponin 1, thornasteroside A, marthasteroside A1, and regularoside A) were identified via further bioassay-guided fractionation of a new n-BuOH extract[6-8]. All asterosaponins exhibited moderate cytotoxicity, with asterosaponin 1 and novaeguinoside II demonstrating the highest level of bioactivity, showing inhibitory concentrations 2–4 times lower than the other saponins tested[6-8]. Later research isolated four more asterosaponins (novaeguinosides A–D) from *C. novaeguineae* and tested their ability to promote tubulin polymerisation *in vitro* as potential antitumor agents the K-562 and BEL-7402 cell lines[54]. All four asterosaponins showed cytotoxicity, with IC_{50} values ranging from 0.7 to 9.5 μM. Novaeguinoside C exhibited the highest potency (IC_{50} of 1.3 and 0.7 μM, respectively), although none of the saponins tested demonstrated the ability to polymerise tubulin[54]. The combined findings from these studies suggested that specific structural traits, such as $\Delta9(11)$-3β,6α-dioxysteroidal aglycone with a C-3 sulfate group and an oligosaccharide moiety at C-6 as a potent configuration, are responsible for potent cytotoxic activity[54].

In one study, nine triterpenoid saponins (pacificusosides D-K and cucumarioside D) isolated from *Solaster pacificus* were tested for cytotoxicity against non-cancerous mouse epidermal cells (JB6 CI41) and a panel of human melanoma cell lines (SK-MEL-2, SK-MEL-28, and RPMI-7951) at concentrations ranging from 0.1 to 62.5 μM for 24 h[15]. The non-cancerous JB6 CI41 served as a reference to assess cancer selectivity, with greater differences in IC50 values between cancer and non-cancer cells indicating higher selectivity[15]. Among the saponins tested, pacificusosides D, F, H, K and cucumarioside D exhibited high selectivity and cytotoxicity specifically against the SK-MEL-2 cell line, with IC_{50} values of 0.7, 0.68, 0.69, 0.75, and 0.67 μM, respectively. In contrast, pacificusosides E, G, I, and J were less effective, displaying equal or higher IC_{50} values compared to the JB6 cells, and overall, the RPMI-7951 cell line showed resistance to the bioactivity of these saponins[15].

Another study explored the cytotoxic properties of three asterosaponins (asteriidoside A, diplasteriosides A and B) isolated from *Diplasterias brucei* against human colon cancer

cells (HCT116), breast cancer cells (T-47D), and melanoma cancer cells (RPMI-7951)[44]. Diplasterioside A exhibited cytotoxicity across all three cell types, with IC_{50} values ranging from 60 µM against RPMI-7951 to 153 µM against T-47D. Diplasterioside B showed toxicity against RPMI-7951 cells (IC_{50} of 67 µM), while asteriidoside A displayed no cytotoxicity at concentrations below 160 µM[44].

Four asterosaponins, acanthaglycoside G, pentareguloside G, acanthaglycoside A and maculatoside, were isolated from an ethanolic extract of *Acanthaster planci* and tested for cytotoxic activity against three human cancer cell lines RPMI-7951, HT-29 (colorectal carcinoma), MDA-MB-231 (breast cancer)[42]. While acanthaglycoside G and pentareguloside G exhibited no significant inhibitory activity, acanthaglycoside A and maculatoside demonstrated effective bioactivity against both HT-29 (IC_{50}: 11 and 7 µM, respectively) and MDA-MB-231 (IC_{50}: 11 and 8 µM, respectively), though they were effective against RPMI-7951 cells (IC_{50}: 15 and 14 µM, respectively). Among the tested asterosaponins, maculatoside showed the highest potential for inhibiting cell colony growth across all three cell lines[42].

The *in vitro* cytotoxicity of four other asterosaponins (lethasterioside A, thornasteroside A, anasteroside A and luidiaquinoside) isolated from *Lethasterias fusca* was assessed against three human cancer cell lines: breast T-47D, colorectal HCT-116, and melanoma RPMI-7951[41]. Thornasteroside A exhibited cytotoxicity across all three cell lines, with IC_{50} values ranging from 50 to 94 µM. Lethasterioside A was nontoxic to the T-47D cell line but showed strong cytotoxicity against HCT-116 and RPMI-7951 cells, with IC_{50} values of 41 and 34 µM, respectively. In contrast, anasteroside A and luidiaquinoside did not demonstrate any bioactivity at concentrations below 160 µM for any of the tested cell lines[41].

Additionally, ten steroidal saponins (certonardosides B2, B3, H2-4, I2, J2, J3, O1, and P1) isolated from *Certonardoa semiregularis* were tested for cytotoxicity against a small panel of human solid tumour cell lines (A549, SK-OV-3, SK-MEL-2, XF498, and HCT15). All but one saponin demonstrated moderate to significant cytotoxicity[49, 51]. Certonardosides P1 and J3 were shown to be the most potent, with ED_{50} values ranging from 0.35 to 2.17 µg/mL and 0.45 to 2.76 µg/mL, respectively. Certonardoside H3 showed considerable cytotoxicity, with ED_{50} values between of 0.52 and 7.20 µg/mL. Notably, the HCT15 cell line demonstrated greater resilience, compared to the other four tumour cell lines, recording the highest effective dosage requirement (8.13, 6.14, 5.04, 7.20, >30, 8.32, 8.20, 2.76, 2.28, and 2.17 µg/mL, respectively) in all but one case[49, 51].

Four asterosaponins–aphelasteroside A, an asterone analogue of thornasteroside A, thornasteroside A, and versicoside A–isolated from *Asterias microdiscus* were tested for cytotoxic effects against four human cancer cell lines: HT-29, MDA-MB-231, THP-1 (monocytic leukemia), and Raji (Burkitt's lymphoma), as well as a normal mouse epidermal cell line (JB6 Cl41), at concentrations up to 100 μM[40]. Aphelasteroside A and the asterone analogue demonstrated cytotoxicity against THP-1 and Raji cells, with IC_{50} values below 100 μM, while the other three cell lines consistently required concentrations greater than 100 μM to achieve similar effects. THP-1 cells were particularly sensitive, with versicoside A achieving the lowest tested IC_{50} value of 23 μM. Thornasteroside A exhibited the least variation in inhibitory concentration across between the tested cell lines, ranging from 28 to 48 μM. The study highlighted that thornasteroside A and versicoside A to be promising candidates for future investigation into their anticancer mechanisms, particularly for breast cancer[40].

Six new asterosaponins, leptasteriosides A–F, were isolated from the alcoholic extract of the Far Eastern starfish *Leptasterias ochotensis* and cytotoxic activities tested against human breast cancer T-47D and melanoma RPMI-7951 cell lines[48]. Leptasteriosides D, E, and F exhibited slight cytotoxic effects against T-47D and against RPMI-7951 cell lines with IC_{50} values of 68 to 143 μM, and 91 to 151 μM, respectively. In contrast, leptasteriosides A, B, and C displayed significant inhibition of both cell lines, with IC_{50} values of 2, 10, and 23 μM against T-47D, and 27, 17 and 30 μM against RPMI-7951, respectively[48].

Eleven polyhydroxysteroidal glycosides (anthenosides A–K) were isolated from an ethanol extract of *Anthenea chinensis* and evaluated for cytotoxicity against human leukemia K-562, hepatoma BEL-7402, and glioblastoma U87 cells[55, 56]. Among the isolated compounds, anthenoside A showed the highest cytotoxicity, with IC_{50} values of 1.62, 3.09, and 2.79 μM across the three cell lines. The greatest bioactivity was observed in a mixture of anthenosides J and K (ratio unknown), which achieved an IC_{50} of 0.60 μM against K-562 cells. The U87 cell line was generally less sensitive, requiring higher IC_{50} value (> 25 μM) for some anthenosides compared to the K-562 and BEL-7402 cell lines, where IC_{50} values ranged between 2.8 to 8.3 μM. The other anthenosides did not exhibit significant cytotoxic effects[55, 56].

The cytotoxic activities of three polyhydroxysteroidal saponins (hesperuside A, B, and C) isolated from the ethanol extract of *Craspidaster Hesperus*, were tested against human tumour cells hepatoma BEL-7402, leukemia MOLT-4, and lung cancer A-549 *in vitro*[53].

Hesperuside B demonstrated the highest cytotoxicity across all tested tumour cells, with IC_{50} values of 2.67, 0.68 and 1.84 μM, respectively. Hesperusides A and C were deemed less potent, with IC_{50} values ranging from 2.12 to 5.72 μM. The MOLT-4 leukemia cells were the most susceptible to hesperuside exposure, requiring the lowest IC_{50} concentrations[53].

Eight steroidal saponins isolated from a methanol extract of *Culcita novaeguineae* were tested for cytotoxic activity against five human cancer cell lines: hepatoma Hep-G2, epidermoid carcinoma KB, prostate LNCaP, breast MCF7, and melanoma SK-Mel2[37]. Halitylosides D demonstrated the strongest cytotoxicity, achieving the lowest IC_{50} values across all five cell lines (75.01, 32.66, 31.80, 33.96, and 32.99 μM, respectively). Halityloside B also showed moderate activity, with IC_{50} values ranging from 39.68 to 80.22 μM. Halityloside A and culcitoside C5 exhibited weaker bioactivity, with IC_{50} values between 48.59 to 92.04 μM for the KB, LNCaP, MCF7, and SK-Mel2 cell lines. Neither compound achieved an IC_{50} below 100 μM for the Hep-G2 cell line. The remaining four saponins did not exhibit significant cytotoxic effects below 100 μM in any of the tested cell lines. Overall, Hep-G2 cells were the most resistant, requiring the highest IC_{50} doses for all tested saponins[37].

The anti-cancer potential of three asterosaponins (regularoside A, archasterosides A, and B) isolated from *Archaster typicus*, was tested against human HeLa and mouse epidermal JB6 Cl41 cells[57]. Among the tested compounds, archasteroside B exhibited the highest potency, with IC_{50} values of 14 and 18 μM against HeLa and JB6 Cl41 cells, respectively. Archasteroside A showed moderate activity, with IC_{50} values of 24 and 37 μM. In contrast, regularoside A displayed only weak bioactivity against HeLa cells, requiring 110 μM to achieve an IC_{50}, and was deemed inactive to JB6 cells at concentrations below 50 μM[57].

In a separate study, a bioassay guided fractionation of a methanolic extract from *Certonardoa semiregularis* led to the isolation of two steroidal saponins, certonardosides P2 and I3. These compounds were tested for cytotoxicity against five human cancer cell lines: A549 (lung), SK-OV-3 (ovary), SK-MEL-2 (melanoma), XF498 (glioblastoma), and HCT15[50]. Certonardoside P2 demonstrated strong cytotoxic activity against the SK-MEL-2 cell line, with an IC_{50} value of 2.67 μM. The other cell lines were less sensitive, with IC_{50} values of 19.5, 26.5, and 18.2 μM for SK-OV-3, XF498, and HCT15, respectively. Certonardoside P2 was not active against A549 cells at concentrations below 30 μg/mL. Certonardoside I3 showed no bioactivity against any of the tested cell lines at concentrations under 30 μg/mL[50].

Moreover, fifteen steroidal saponins (certonardosides A-N and culcitoside C6), were isolated from a methanol extract of *Certonardoa semiregularis*, guided active in a brine shrimp assay[36]. These saponins were evaluated for cytotoxicity against a panel of five human solid tumour cell lines, including A549 lung, SK-OV-3 ovary, SK-MEL-3 melanoma, XF498 Glioblastoma and HCT15 colorectal. Certonardosides L and N demonstrated considerable cytotoxicity against all five cell lines, with effective dosages (ED_{50}) between 3.9 to 8.4 µg/mL. Certonardoside C showed the highest bioactivity, with an ED_{50} value of 3.8 µg/mL against SK-MEL-2, although it displayed only moderate cytotoxicity against the other four cell lines, with ED50 values ranging from 15.8 to 25.9 µg/mL ED_{50}. Furthermore, certonardosides A, H, K, M, and culcitoside C6 demonstrated moderate to strong bioactivity against the SK-MEL-2 cell line, with ED_{50} values between 6.7 to 16.3 µg/mL. Their effects on the other four cell lines, however, were limited to non-existent. Certonardosides B, D-G, I, and J showed no significant bioactivity for any cell line tested below 30 µg/mL. Overall, SK-MEL-2 appeared distinctly more susceptible to saponin exposure compared to the other tested cell lines[36].

Eight steroidal saponins (certonardosides A, C, D, F, H-J, and halityloside D), isolated from the brine shrimp active fraction of a methanolic extract of *Certonardoa semiregularis*, were evaluated for their potential antiviral activity and cytotoxicity using three host cell lines: African green monkey (*Chlorocebus sabaeus*) Vero cells, human leukemia MT-4, and human cervical HeLa cell lines[38]. Although the saponins exhibited only weak antiviral activity, the cytotoxicity tests revealed notable anti-cancer potential. Certonardosides A and C were particularly cytotoxic against the MT-4 cell line, with IC_{50} values of 6.8 and 1.4 µM, respectively. However, these compounds had limited effects on the other cell lines, with only Certonardoside C showing weak cytotoxicity against the Vero cells, achieving an IC_{50} of 97.5 µM. The remaining six saponins demonstrated moderate cytotoxicity against the tested cell lines, with IC_{50} values ranging from 39.7 to 79.9 µM. Each of these six saponins was selective for one or two cell lines, while exhibiting no effect on the third[38].

Six steroidal saponins isolated from a methanolic extract of *pentaceraster gracilis*, were evaluated for cytotoxicity against five human cancer cell lines including Hep-G2, KB, LNCaP, MCF7, and SK-MEL-2[43]. Maculatoside showed significant cytotoxic effect against the Hep-G2 and SK-Mel2 cell lines, with IC_{50} values of 16.75 and 19.44 µM, respectively. Moderate effect was observed on KB, LNCaP and MCF7 with 36.53, 39.75 and 47.34 µM. The other five saponins showed no cytotoxicity at concentrations below 100 µM against any of the tested cell lines[43].

Three steroidal saponins (polyacanthoside A, marthasteroside B, and psilasteroside) isolated from the methanolic extract of *Astropecten polyacanthus* were tested for cytotoxic activity against five human cancer cell lines: HepG2, KB, LNCaP, MCF7, and SK-Mel2[52]. Marthasteroside B and psilasteroside exhibited moderate cytotoxic effects across all five cell lines, with IC_{50} values ranging from 28.71 to 49.03 μM. However, polyacanthoside A showed no bioactivity at concentrations below 100 μM[52].

The cytotoxicity of six triterpenoid saponins (pacificusosides A-C and cucumariosides C1, C2, and A10) isolated from an alcoholoic extract of *Solaster pacificus* was tested against human embryonic kidney HEK 293, colorectal carcinoma HT-29, melanoma RPMI-7951, and breast MDA-MB-231 cell lines[28]. All three cucumarioside saponins exhibited strong cytotoxicity against all tested cell lines, with IC_{50} values ranging from 3.6 to 7.9 μM. Pacificusoside C was similarly cytotoxic to three of the four cell lines, with IC_{50} values between 6.2 and 6.7 μM, though it was less effective against RPMI-7951, with an IC_{50} value of 23.7 μM. Pacificusoside B demonstrated moderate cytotoxicity to all cell lines, with IC_{50} values between 20.4 and 28.6 μM, whereas pacificusoside A showed no cytotoxicity below 40 μM[28].

Seven steroidal saponins (cariniferoside A and F; halityloside A, B, and D; halityloside A 6-O-sulfate, and 4-O-methyl halityloside A 6-O-sulfate), isolated from *Asteropsis carinifera*, were tested for *in vitro* cytotoxicity against human cancer cell lines HCT-116, T-47D, and RPMI-7951[39]. Of these, cariniferoside A demonstrated moderate cytotoxicity against all tested cell lines, with IC_{50} values of 32, 37, and 66 μM, respectively. Halityloside A and B showed weak bioactivity, with halityloside A active only against HCT-116 (IC_{50} of 150 μM) and halityloside B active only against T-47D and RPMI-7951 cells, with IC_{50} values of 154 and 128 μM, respectively[39].

The cytotoxic activities of three steroidal saponins (anthenosides A, A1, and A2) isolated from *Anthenea aspera* were evaluated against human melanoma RPMI-7951 and breast cancer T-47D cells[47]. All three anthenoside saponins demonstrated weak inhibition of T-47D cell viability, with IC_{50} values of 133, 139, and 158μM, respectively. However, none of the saponins displayed cytotoxic effects against RPMI-7951 cells at concentrations below 150 μM[47].

Four steroidal saponins (echinasteroside B, granulatoside A, linckoside K and forbeside L) isolated from *Mithrodia clavigera* were tested for cytotoxic activity against human skin melanoma cells SK-MEL-28, SK-MEL-5, and RPMI-7951[46]. Forbeside L demonstrated the

most significant cytotoxicity among the tested compounds, with moderate effects at IC_{50} values of 75, 79, and 84 μM against the three cell lines, respectively. Granulatoside A and linckoside K exhibited weak cytotoxic activity, with IC_{50} values ranging from 83 to 164 μM. In contrast, echinasteroside B showed no cytotoxic activity at concentrations below 200 μM[46].

Three steroidal saponins (plancisides A–C) isolated from the ethanolic extract of *Acanthaster planci* were tested for *in vitro* cytotoxicity against human colon cancer HCT-116, breast cancer T-47D and melanoma RPMI-7951 cells[45]. Planciside A exhibited moderate cytotoxicity against HCT-116 and RPMI-7951 cells, with IC_{50} values of 36 and 58 μM, respectively, but showed no toxicity below 120 μM against the T-47D cell line. Planciside B and C showed no cytotoxic activity at any concentration tested with these three cell lines[45].
